# The Regulatory Effect of UL-16 Binding Protein-3 Expression on the Cytotoxicity of NK Cells in Cancer Patients

**DOI:** 10.1038/srep06138

**Published:** 2014-08-20

**Authors:** Xiao Mou, Yuepeng Zhou, Peng Jiang, Tong Zhou, Qian Jiang, Chengcheng Xu, Hongli Liu, Tingting Zheng, Guoyue Yuan, Yanyun Zhang, Deyu Chen, Chaoming Mao

**Affiliations:** 1 Department of Nuclear Medicine, The Hospital Affiliated to Jiangsu University, Zhenjiang, China; 2 Institute of Oncology, The Hospital Affiliated to Jiangsu University, Zhenjiang, China; 3 Department of anesthesiology, The Hospital Affiliated to Jiangsu University, Zhenjiang, China; 4 Department of Pediatrics, Ruijin Hospital, Shanghai Jiao Tong University School of Medicine, Shanghai, China; 5 Key Laboratory of Stem Cell Biology, Institute of Health Sciences, Shanghai Institutes for Biological Sciences, Chinese Academy of Sciences, Shanghai, China; 6 These authors contributed equally to this work.

## Abstract

The activating immunoreceptor NKG2D (natural killer group 2, member D) and its ligands play important roles in the innate and adaptive immune responses. UL16-binding protein 3 (ULBP3), an NKG2D ligand, is overexpressed on certain epithelial tumor cells. In this study, we investigated the effect of ULBP3 expression on the cytotoxic activity of natural killer (NK) cells. ULBP3 were measured by flow cytometry analysis, immunohistochemistry, and time-resolved fluoroimmunoassay. The cytotoxicity of NK cells was determined with the lactate dehydrogenase release assay. We found that ULBP3 was overexpressed on tumor cell lines and tumor tissues. Serum from cancer patients, but not from healthy donors, contained elevated levels of soluble ULBP3 (sULBP3). Importantly, high expression of ULBP3 on the cell surface of tumor cells augmented NKG2D-mediated NK cell cytotoxicity. However, low levels of sULBP3 (<15 ng/ml) weakened the cytotoxicity of NK cells by decreasing NKG2D expression on NK cells. Further analysis showed that serum samples from most cancer patients (>70%) contained the low level of sULBP3. Our results demonstrate that tumor cells express surface and soluble ULBP3, which regulate NK cell activity. Thus, ULBP3 is a potential therapeutic target for improving the immune response against cancer.

Natural killer (NK) cells, components of the innate immune system, contribute to the elimination of virus-infected cells as well as to antitumor immune responses^1^. NK cell reactivity is guided by the principles of “missing-self” and “induced-self,” in which NK cells are activated by the downregulation or absence of major histocompatibility complex (MHC) Ι expression (“missing-self”) and/or by the stress-induced expression of ligands that bind activating NK receptors (“induced-self”). The balance of various activating and inhibitory signals determines whether NK cell responses are initiated^2^,^3^,^4^,^5^.

Among the activating NK receptors, NKG2D (natural killer group 2, member D) is particularly relevant for tumor cell recognition and killing. NKG2D is a C-type lectin-like activating receptor expressed on the cell surface of almost all NK cells, some cytotoxic CD8^+^ αβ T cells, NK T cells, and γδ T cells, and a small subset of CD4^+^ αβ T cells^6^,^7^,^8^. NKG2D mediates NK cell activation by overcoming inhibitory signals from self recognition^9^,^10^. Malignant transformation induces the expression of NKG2D ligands (NKG2DL), as documented in a variety of human and mouse tumors. The activating immunoreceptor NKG2D endows cytotoxic lymphocytes with the capacity to recognize and eliminate malignant cells, and it plays a critical role in immune surveillance^11^. For example, NKG2DL-expressing tumor cells grafts were efficiently rejected, whereas parental NKG2D-ligand negative tumor cells formed tumors^12^,^13^. A distinctive feature of the NKG2D recognition system is that NKG2D can interact with a number of distinct ligands with affinities ranging from 4 to 400 nM^14^,^15^,^16^. The ligands recognized by NKG2D, which belong to distinct and relatively distantly related families, include major histocompatibility complex class-I related chain (MIC) A, MICB, and UL16-binding proteins (ULBPs) in humans^10^,^17^. NKG2DLs are generally not expressed on benign cells, but are induced by cellular stress, genotoxic stress, and infection^18^,^19^. The human ULBP proteins are widely expressed by various tumor types, including leukemia, and primary solid tumors^20^,^21^,^22^.

In addition to expressing NKG2DLs on their surface, tumors spontaneously release soluble ligands^23^. Soluble MICA secreted by tumor cells downregulated surface NKG2D expression on T cells to induce the functional impairment of anti-tumor immune effector cells, suggesting that shedding may reduce the expression of NKG2DLs on the tumor cell surface and contribute to tumor escape from immunosurveillance. Soluble MICA induced the internalization and lysosomal degradation of the NKG2D receptor in CD8^+^ T and NK cells^24^,^25^,^26^, further reducing the efficiency of NKG2D recognition. Elevated serum levels of soluble MICA have been detected in patients with various types of cancer and may represent a diagnostic marker in patients with suspected malignancies^27^,^28^.

Unlike other NKG2DLs, ULBP3 has a moderate affinity for NKG2D. However, the regulatory function of ULBP3 in NK cells and its significance in cancer patients are largely unknown. In the present study, ULBP3 expression in several tumor cell lines and tumor tissue cells from common cancer types was analyzed. The effects of surface and soluble forms of ULBP3 on the interaction between tumor cells and NK cells were examined. Our results showed that ULBP3 regulated the activity of NK cells against tumors. Thus, ULBP3 provides a target for tumor immunotherapy.

## Results

### Elevated expression of ULBP3 in tumor cell lines and tumor tissues

**Figure 1:**
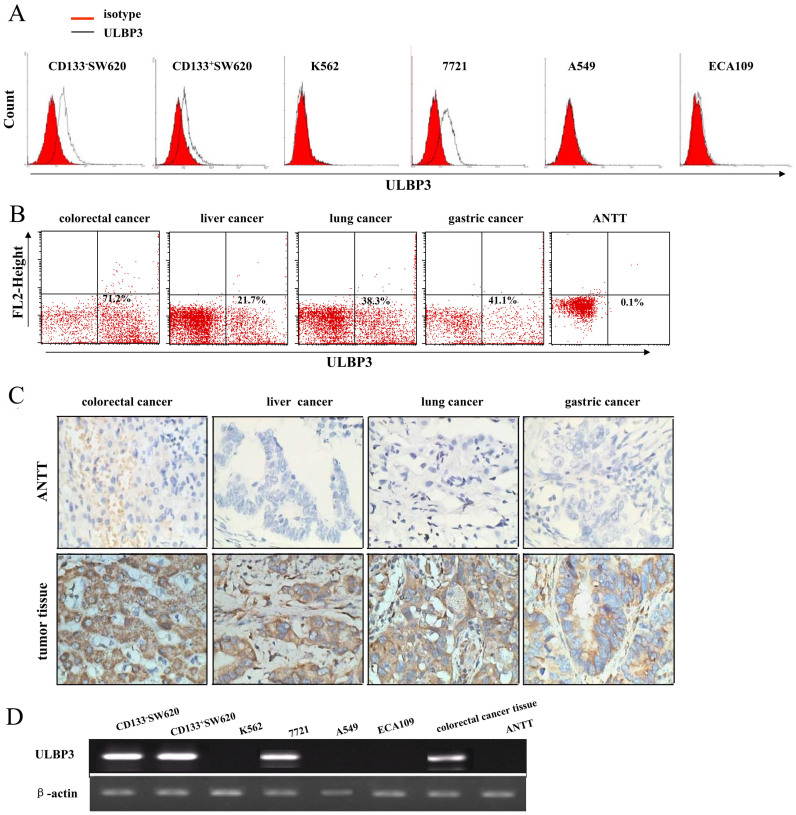
ULBP3 expression in tumor cell lines and tumor tissues. (A) Surface expression of ULBP3 on CD133^−^SW620, CD133^+^SW620, K562, 7721, A549, and ECA109 cell lines was measured by flow cytometry (FCM) (n = 3). ULBP3 protein was detected with the monoclonal antibody B2-F1-F1 (5 μg/ml) and a FITC-conjugated goat anti-mouse antibody. On each histogram, the red regions represent the isotype controls, and the black lines represent the marker of interest. (B) ULBP3 expression in single-cell suspensions of fresh tumor tissues from colorectal cancer (n = 3), liver cancer (n = 3), lung cancer (n = 3), and gastric cancer (n = 3) and paired adjacent non-tumor tissues (ANTT) was detected by FCM. Representative dot graphs are shown. The results are expressed as the mean ± SEM. (C) Representative results from immunohistochemical staining for ULBP3 in colon, liver, lung, and stomach tumor tissues and ANTT are shown. Brown regions represent positive expression of ULBP3. Control indicates ANTT. The slides were analyzed under a 10 × 40 microscope equipped with a camera. (D) Total mRNA was extracted from tumor cell lines, colorectal tumor tissues and ANTT using TRIzol reagent. The mRNA expression of ULBP3 and β-actin was evaluated using RT-PCR. PCR products were visualized by gel electrophoresis in the presence of ethidium bromide. The data are representative of results obtained from at least 3 independent experiments.

To evaluate the distribution of the NKG2DL ULBP3 in tumor cells from common cancers, the surface expression of ULBP3 in SW620, K562, 7721, A549, and ECA109 cell lines was analyzed by flow cytometry (FCM) analysis. The colorectal cancer cell line CD133^−^SW620 expressed high levels (>50%) of ULBP3 (59.0 ± 2.6%, n = 3), and CD133^+^SW620 cells expressed moderate levels (20%–50%) of ULBP3 (22.0 ± 1.4%, n = 3). The liver cancer cell line 7721 also expressed a moderate level of ULBP3 protein (30.0 ± 3.7%, n = 3). However, surface ULBP3 protein was undetectable on the lung cancer cell line A549 and esophageal carcinoma cell line ECA109. The leukemic cell line K562, which does not express surface ULBP3, was used as a negative control (Figure 1A). We then examined the expression of ULBP3 in different tumor tissues. In cancer patients with colorectal cancer (n = 5), liver cancer (n = 3), lung cancer (n = 3), and gastric cancer (n = 7), FCS indicated that ULBP3 expression was much higher in the tumor tissue than in the adjacent non-tumor tissue (ANTT). Representative dot graphs are shown in Figure 1B. Immunohistochemistry results also showed that ULBP3 expression was upregulated in colorectal (n = 5), liver (n = 3), lung (n = 5), and gastric (n = 5) cancer tissue (Figure 1C). The results of RT-PCR analysis demonstrated that the *ULBP3* gene was expressed in ULBP3-expressing tumor cell lines and in fresh tumor tissues from cancer patients, but not in ANTT (Figure 1D).

### Spontaneous release of soluble ULBP3 (sULBP3) from tumor cell lines and elevated serum sULBP3 in cancer patients

**Figure 2:**
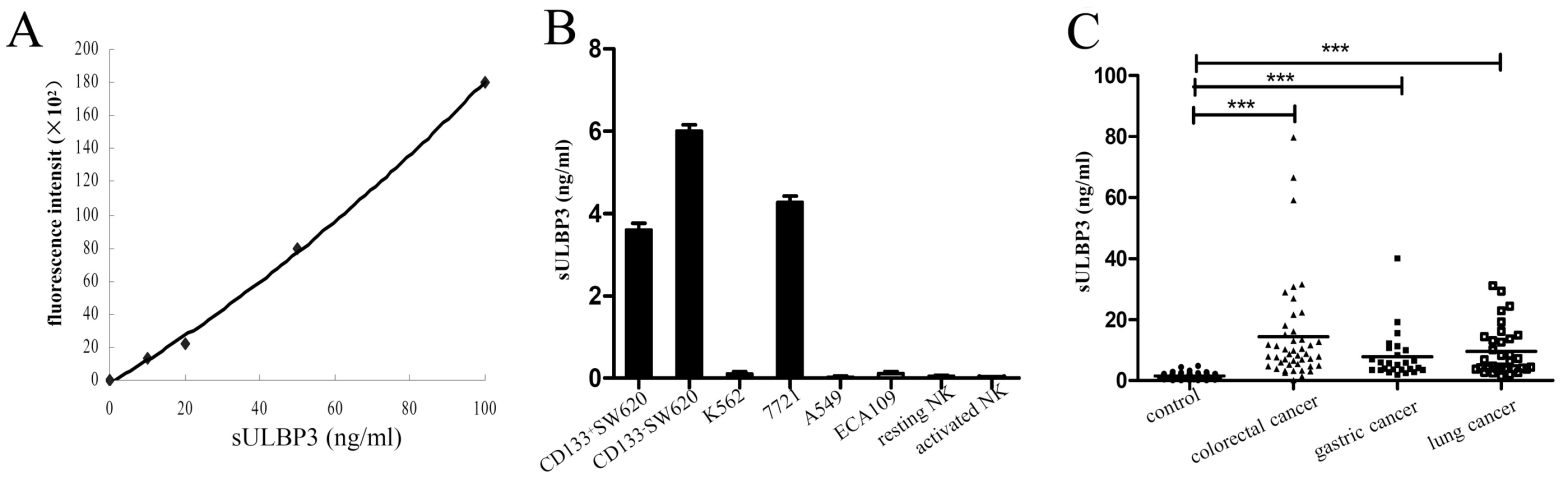
Detection of soluble ULBP3 (sULBP3) from tumor cells and in the serum of cancer patients. sULBP3 in serum samples from cancer patients or in the culture supernatant of tumor cell lines was measured by time-resolved fluoroimmunoassay (TRFIA), as described in the Materials and Methods section. (A) Standard curve for the detection of the sULBP3. (B) sULBP3 levels in the culture supernatant of cancer cell lines. (C) sULBP3 levels in the serum of healthy volunteers (n = 48) and patients with colorectal caner (n = 45), gastric cancer (n = 38), or lung cancer (n = 33). The results are expressed as the mean ± SEM. ***P < 0.001.

To determine whether tumor cells release sULBP3, we used a time-resolved fluoroimmunoassay (TRFIA) with a Eu^3+^-labeled anti-ULBP3 monoclonal antibody. The detection limit of the TRFIA method for ULBP3-Fc was approximately 0.05 ng/ml (Figure 2A). After 48 h of culture, sULBP3 levels in the supernatants of SW620, K562, 7721, A549, and ECA109 cells were measured. No sULBP3 was detectable in the culture supernatants of K562, A549, and ECA109 cells or in the culture supernatants of resting or activated NK cells. However, the levels of sULBP3 were detected in the supernatants of the tumor cell lines CD133^+^SW620 (3.6 ± 0.08 ng/ml), CD133^−^SW620 (6.0 ± 0.1 ng/ml), and 7721 (4.3 ± 0.1 ng/ml) (Figure 2B). To determine whether ULBP3 was released by human tumors in vivo, we analyzed serum sULBP3 levels in 116 patients with various malignancies and 48 healthy volunteers. Compared with healthy volunteers (1.5 ± 0.1 ng/ml), the serum concentration of sULBP3 was significantly higher in cancer patients (colorectal cancer: n = 45, 14.4 ± 2.5 ng/ml, *P* < 0.001; gastric cancer: n = 38, 8.4 ± 1.1 ng/ml, *P* < 0.001; lung cancer: n = 33, 9.6 ± 1.4 ng/ml, *P* < 0.001) (Figure 2C). Further analysis showed that 73.3% of colorectal cancer patients, 83.3% of gastric cancer patients, and 82.3% of lung cancer patients were distributed in the low level of serum ULBP3 (<15 ng/ml), as shown in Table 1.

### Differences in the functional effects of surface ULBP3 and sULBP3 on NK cell activity

**Figure 3:**
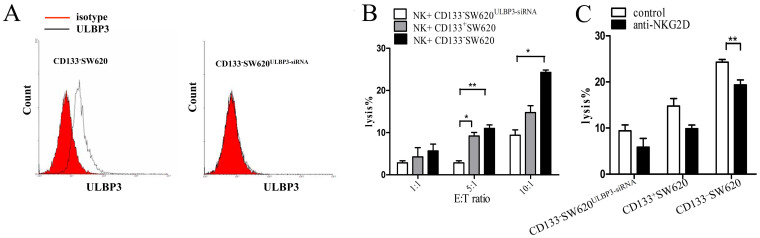
The effect of surface-expressed ULBP3 on NK cell activity. (A) The surface expression of ULBP3 on CD133^−^SW620 cells (left) or CD133^−^SW620 cells with ULBP3-siRNA transfection (right) was measured by FCM. (B–C) Effector NK cells were freshly isolated from healthy volunteers (normal NK cells), and 3 cell lines (ULBP3 siRNA-treated CD133^−^SW620, CD133^+^SW620, and CD133^−^ SW620) were used as target cells. The cytotoxicity of the NK cells was measured by LDH release after co-culture for 6 h at the indicated effector cell to target cell (E:T) ratios (B) and with or without the addition of anti-NKG2D (5 μg/ml) in co-cultures with an E:T ratio of 10:1 (C). The data are representative of results obtained from at least 3 independent experiments. The results are expressed as the mean ± SEM. *P < 0.05, **P < 0.01.

Next, we assessed the effect of ULBP3 cell surface expression on the lytic capacity of effector NK cells. CD133^+^SW620 (moderate expression of surface ULBP3), CD133^−^SW620 (high expression of surface ULBP3), and CD133^−^SW620^ULBP3-siRNA^ (no/low expression of surface ULBP3; Figure 3A) tumor cells were used as target cells, and NK cells were freshly isolated from healthy volunteers for use as effector cells in cytotoxicity assays. With CD133^−^SW620^ULBP3-siRNA^ cells, the efficiency of NK-cell mediated killing was low when the effector cell to target cell (E:T) ratio was low (5:1 or 1:1) or high (10:1). However, a high level of cytotoxicity was observed with CD133^−^SW620 cells, and a moderate level of cytotoxicity was found with CD133^+^SW620 cells. These results suggested that the amount of ULBP3 expressed on the cell surface of tumor cells affected the cytotoxicity of NK cells (Figure 3B). In these experiments, blocking NKG2D on the surface of NK cells with a specific neutralizing monoclonal antibody strongly reduced the cytolytic activity of NK cells against the target cells at an E:T ratio of 10:1, indicating that the NKG2D/NKG2DL pathway was involved in modulating the cytotoxic activity of NK cells (Figure 3C).

**Figure 4:**
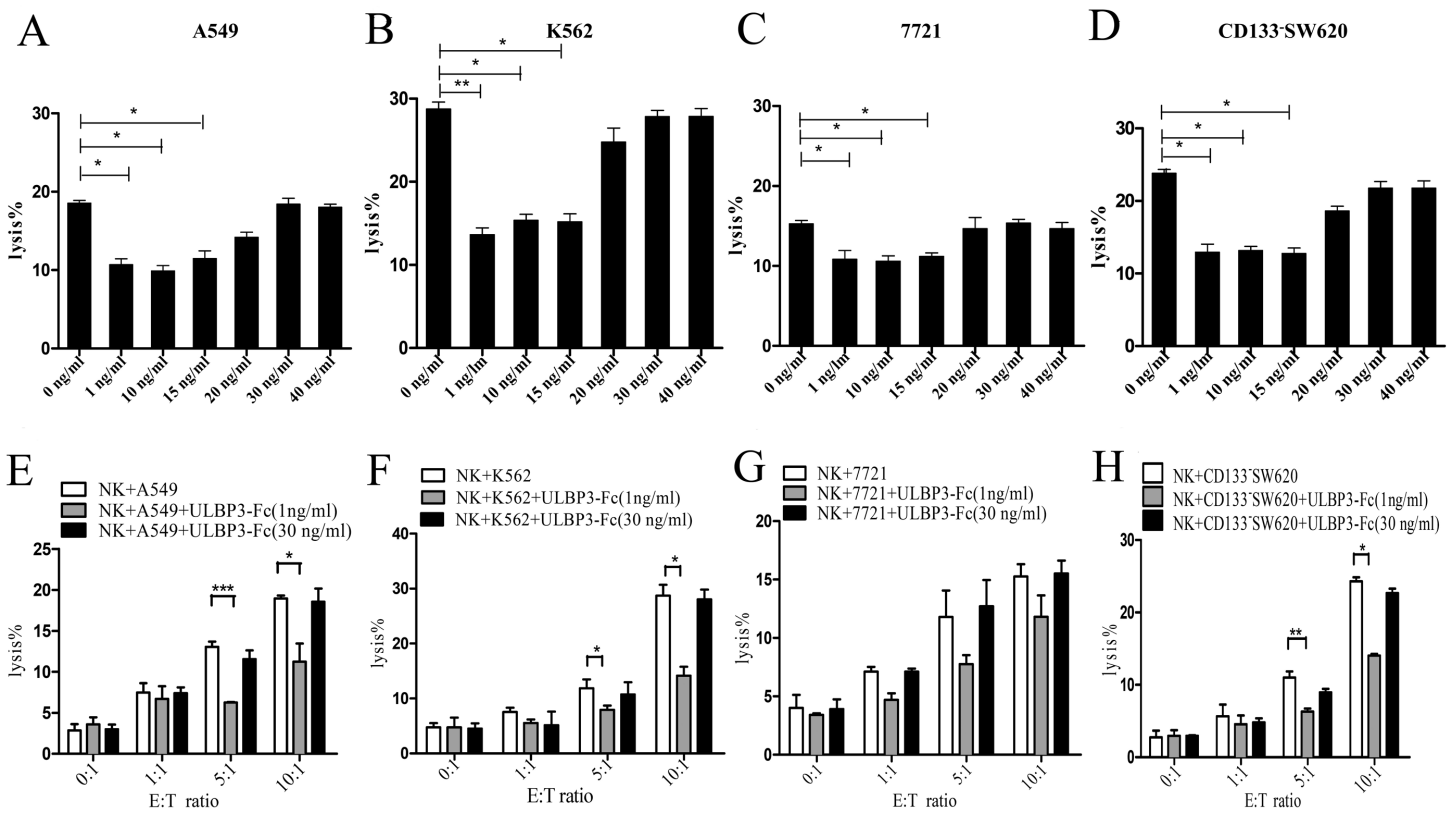
The effect of sULBP3 on NK cell activity. A549 (A), K562 (B), 7721 (C), and CD133^−^SW620 (D) cells were co-cultured with NK cells, with or without the indicated concentrations of ULBP3-Fc. NK cell cytotoxicity was measured by LDH release after co-culture for 6 h at an E:T ratio of 10:1. A549 (E), K562 (F), 7721 (G), and CD133^−^SW620 (H) were co-cultured with NK cells respectively at the indicated E:T ratios, with or without adding high (30 ng/ml) or low (1 ng/ml) concentration of ULBP3-Fc. The NK cell cytotoxicity was measured by LDH release after co-culture for 6 h. Data are representative of the results obtained from at least three independent experiments. Results are expressed as means ± SEM. *P < 0.05, **P < 0.01, ***P < 0.001.

To determine the effect of sULBP3 on the lytic capacity of NK cells, different concentrations of soluble recombinant ULBP3-Fc were added to the culture medium of tumor cells and NK cells, and cytotoxicity was measured. In the absence of surface ULBP3 expression on K562 and A549 cells, the addition of a low dose of sULBP3 (<15 ng/ml) reduced NK cell-mediated cytotoxicity against K562 cells at an E:T ratio of 10:1. However, no significant increase in NK cell cytotoxicity was observed when high concentrations of sULBP3 (>15 ng/ml) were added to co-culture of NK and target cells (Figure 4A and 4B). With 7721 and CD133^−^SW620 cells expressing moderate to high levels of surface ULBP3, similar results were observed (Figure 4C and 4D). Then, we studied the effect of soluble ULBP3 on the lytic capacity of NK cells on different E:T ratios, we found that, firstly, in the non-expression of surface ULBP3 on K562 and A549 target cells, the presence of low dose of sULBP3 (1 ng/ml) caused a statistically reduction of NK cell cytotoxicity against target cells at E:T ratio of 5:1 and 10:1. However, no significant increase of NK cell cytotoxicity was observed when high concentration of sULBP3 (30 ng/ml) was added in co-culture of NK and target cells (Figure 4E and 4F). Secondly, in the moderate/high expression of surface ULBP3 on 7721 and CD133^−^SW620 target cells, the similar results were observed (Figure 4G and 4H).

**Figure 5:**
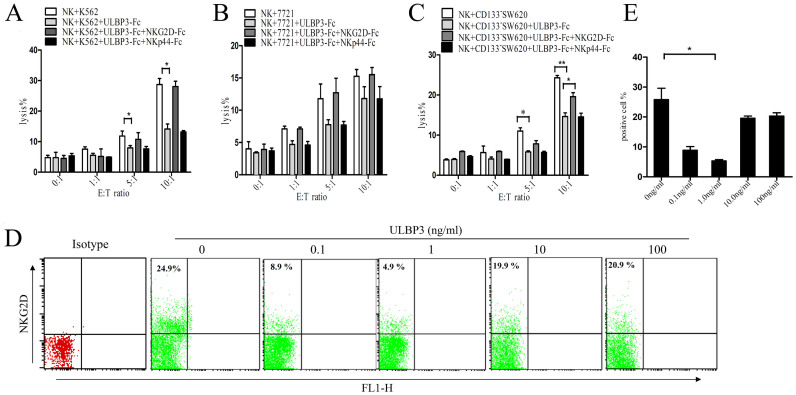
NKG2D reverses the sULBP3-induced downregulation of NK cell cytotoxicity. K562 (A), 7721 (B), or CD133^−^SW620 (C) cells were co-cultured with normal NK cells, with or without ULBP3-Fc (1 ng/ml), NKG2D-Fc (5 ng/ml), and NKp44-Fc (5 ng/ml). The cytotoxicity of NK cells was measured by LDH release after co-culture for 6 h at the indicated E:T ratios. (D) Normal NK cells were incubated with the indicated concentrations of ULBP3-Fc for 6 h, and NKG2D expression in NK cells was measured by FCM. A representative graph of NKG2D expression is shown. (E) Combined data of NKG2D expression are shown. The data are representative of results obtained from at least 3 independent experiments. The results are expressed as the mean ± SEM. *P < 0.05, **P < 0.01.

To confirm that sULBP3 regulated NK cell-mediated cytotoxicity, we incubated sULBP3-containing supernatants for 30 min in the absence or presence of an NKG2D-Fc fusion protein (5 ng/ml) before adding the supernatants to co-culture of NK cells and tumor cells. Neutralization of sULBP3 by NKG2D-Fc diminished the inhibitory effect of the sULBP3-containg supernatants on NK cell cytotoxicity against K562 (Figure 5A), 7721 (Figure 5B), and CD133^−^SW620 (Figure 5C) cells, and no significant effects were detected in the NKp44-Fc control group.

To determine the mechanism by which sULBP3 affects the activity of NK cells, NK cells were pre-incubated with different concentrations of ULBP3-Fc for 6 h, and NKG2D expression was measured. When ULBP3-Fc was added to the culture medium, NKG2D expression on NK cells decreased, particularly with 1 ng/ml ULBP3-Fc. A representative graph depicting NKG2D expression is shown in Figure 5D, and combined data are shown in Figure 5E.

### An anti-ULBP3 monoclonal antibody enhances the activity of NK cells from cancer patients via antibody-dependent cell-mediated cytotoxicity (ADCC)

**Figure 6:**
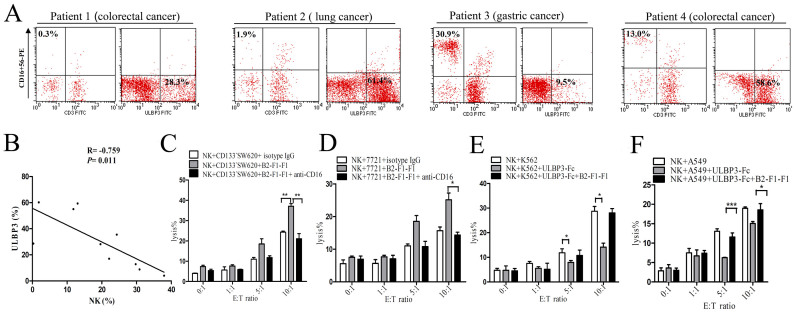
Anti-ULBP3 monoclonal antibody enhances the activity of NK cells from cancer patients via ADCC. ULBP3 expression and the percentage of infiltrating NK cells in tumor tissues from 10 patients (4 colorectal cancer, 3 lung cancer, and 3 gastric cancer) were determined by FCM. A representative graph is shown in (A). The relationship between ULBP3 expression and infiltrating NK cells in tumor tissues is shown in (B). Normal NK cells were co-cultured with CD133^−^SW620 (C) or 7721 (D) cells at the indicated E:T ratios, and cytotoxicity was measured by LDH release after 6 h of co-culture, with or without the addition of isotype IgG, anti-ULBP3 (B2-F1-F1), and/or anti-CD16 (5 μg/ml). K562 (E) or A549 (F) cells were co-cultured with normal NK cells, and cytotoxicity was measured by LDH release after 6 h of co-culture at the indicated E:T ratios, with or without the addition of ULBP3-Fc (1 ng/ml) and/or B2-F1-F1 (5 μg/ml). The data are representative of results obtained from at least 3 independent experiments. The results are expressed as the mean ± SEM. *P < 0.05, **P < 0.01, ***P < 0.001.

To investigate the relationship between the level of ULBP3 expression on tumor cells and the number of infiltrating NK cells in cancer tissues, we measured ULBP3 levels and the percentage of NK cells in tumor tissues from 10 cancer patients (4 colorectal cancer, 3 lung cancer, and 3 gastric cancer). The percentage of infiltrating NK cells negatively correlated with ULBP3 expression (R = −0.759, *P* = 0.011). Representative data from 4 patients are shown in Figure 6A, and combined data are shown in Figure 6B. We then compared NK cytotoxicity in healthy individuals and in cancer patients with low (<15 ng/ml) or high (>15 ng/ml) levels of sULBP3. Freshly isolated NK cells from the peripheral blood mononuclear cells (PBMCs) of healthy individuals (n = 3) and pre-therapy cancer patients (3 colorectal cancer, 2 lung cancer, and 3 gastric cancer) were used as effecter cells. K562 cells served as target cells. The cytotoxicity of NK cells from cancer patients with a low level of sULBP3 were decreased (n = 5, lysis% = 12.3 ± 3.6%, *P* < 0.05), compared with healthy individuals (lysis% = 27.9 ± 2.4%). However, the cytotoxicity of NK cells from cancer patients with a high level of sULBP3 were normal (n = 3, lysis = 25.4 ± 3.7%).

Given that tumor cells generally express surface ULBP3 and sULBP3, we investigated whether an anti-ULBP3 antibody altered the cytotoxic activity of NK cells against ULBP3-expressing tumor cells. The lysis efficiency of the tumor cell lines CD133^−^SW620 (Figure 6B) and 7721 (Figure 6C) at an E:T ratio of 10:1 was enhanced in the presence of anti-ULBP3 monoclonal antibody (B2-F1-F1). Furthermore, when an antibody against CD16 receptor was added, the effect was blocked, suggesting that the elevated activity of NK cells resulted from ADCC. Consistently, the downregulation of NK cytotoxicity by sULBP3 in K562 (Figure 6D) or A549 (Figure 6E) cells lacking ULBP3 was also reversed by B2-F1-F1. Together, these results suggested that B2-F1-F1 abrogated the sULBP3-induced inhibition of NK cell cytotoxicity and induced surface ULBP3-mediated ADCC to enhance the cytotoxicity of NK cells.

## Discussion

By regulating the innate and adaptive immune response through ligation of NKG2D, NKG2DLs are thought to be important for tumor initiation and development. For example, NKG2DL expression has been associated with prognosis, immunoediting, and the control of an immunological checkpoint that relies on NKG2D-mediated immune responses during the epithelial-to-mesenchymal transition^29^,^30^. NKG2DL expression is of particular interest because previous experiments demonstrated that the NKG2D/NKG2DL system stimulates the immune surveillance of tumors^12^,^31^. Thus, NKG2DLs are potential targets for the development of novel immunotherapeutic anticancer regimens^11^. Although NKG2DLs such as MICA/B are widely expressed in many epithelial tumors and are associated with the regulation of NK and γδ T cells, which play a key role in the elimination of tumor cells^32^, other NKG2DLs, such as ULBP1, ULBP2, and ULBP3, are not well characterized. In the present study, we demonstrated that ULBP3, like other NKG2DLs, was widely expressed on primary tumor cells and tumor cell lines. The NK cell cytotoxicity elicited by surface ULBP3-expressing tumor cells was stronger than that elicited by non-expressing tumor cells, suggesting that the expression of surface ULBP3 on tumor cells is directly related to the activity of NK cells against tumor cells.

Previous studies have demonstrated that the shedding of sULBPs can alter the density of surface-expressed NKG2DLs. The soluble molecules have systemic functions that affect NK cell reactivity against tumor cells^33^. Relative to their surface-expressed counterparts, sULBPs may mediate similar, partially different, or opposite effects. In the present study, we demonstrated that ULBP3 was spontaneously released in soluble form from ULBP3-expressing cells. In a high proportion of the serum samples obtained from cancer patients, sULBP3 levels were elevated, indicating that the release of sULBP3 was not an artifact of in vitro tumor cell culture but a feature of human tumors. However, our findings differ from those of previous reports describing the effect of soluble NKG2DL on NK cell activity. In our study, only a low dose of sULBP3 (<15 ng/ml) decreased NK cytotoxicity by downregulating NKG2D expression on NK cells; a high dose of sULBP3 did not inhibit NK cell activity. These findings that a low concentration of sULBP3 downregulated NK cell activity in vitro were consistent with the discovery that more than 70% of cancer patients had a low concentration (<15 ng/ml) of serum sULBP3. Furthermore, the percentage of infiltrating NK cells negatively correlated with ULBP3 expression. These results suggest that a low concentration of sULBP3 contributes to the hyporesponsiveness of the immune system in cancer patients by decreasing NK cell activity, and they explain a general aspect of immunological dysfunction in cancer patients.

Serum levels of soluble MICA/B are elevated in patients with various types of cancer. Soluble MICA/B may be a diagnostic marker in patients with malignancies^27^,^28^. Soluble ULBP2 has also been detected in the serum of some patients with malignancies^34^. We have developed a method for detecting serum ULBP3. Our data demonstrated that the concentration of sULBP3 was significantly higher in colorectal, lung, and gastric cancer patients than in healthy individuals, Furthermore, cancer patients released a significant amount of sULBP3 in vivo. In future studies, it will be important to determine whether the level of serum ULBP3 in cancer patients correlates with the progression and outcome of malignancies, in order to assess ULBP3 as a potential tumor marker.

When analyzing tumor tissue samples from cancer patients, we found a negative correlation between the percentage of infiltrating NK cells and the level of ULBP3 expression. Surface-expressed and soluble ULBP3 may have additive/synergistic effects and cooperate to reduce NK cell reactivity, thus enabling human tumor cells to evade NK-mediated surveillance. Given that tumor cells generally expressed surface ULBP3 and sULBP3, an anti-ULBP3 antibody could contribute to the activity of NK cells against ULBP3-expressing tumor cells. The neutralization of sULBP3 in patients might be a therapeutic strategy to enhance the immune response by promoting NK cell reactivity. Intriguingly, the addition of an anti-ULBP3 antibody prepared in our laboratory triggered ULBP3-mediated ADCC and increased NK cytotoxicity against ULBP3-expressing tumor cells, suggesting the antibody has immunotherapeutic value for cancer treatment.

## Methods

### Patient characteristics and clinical samples

The patients in this study were recruited from The Hospital Affiliated to Jiangsu University and were diagnosed through standard clinical and laboratory examinations. Healthy volunteers were selected based on normal physical and tumor marker examination. Peripheral blood samples were obtained from patients before therapy. Serum was frozen at −80°C for subsequent analysis. Fresh tumor tissue and ANTT were obtained during surgery from cancer patients before they received treatment. The tissue was cleaved and digested with collagenase and pancreatin. Single tumor cell suspensions were washed with PBS and resuspended. All patients and healthy volunteers provided written informed consent in accordance with the guidelines of the Ethics Committee of The Hospital Affiliated to Jiangsu University. This study has been approved by the Ethics Committee of The Hospital Affiliated to Jiangsu University, and was performed accordance with the ethical standards laid down in the 1964 declaration of Helsinki and all subsequent revisions.

### Cell culture and purification

The human tumor cell lines CD133^+^SW620, CD133^−^SW620 (colorectal cancer line), K562 (erythroleukemia cell line), 7721 (liver cancer cell line), A549 (lung cancer cell line), and ECA109 (esophageal carcinoma cell line) were cultured in RPMI 1640 (Life Technologies, Gaithersburg, MD, USA) supplemented with 10% fetal bovine serum (FBS) at 37°C in an atmosphere containing 5% CO_2_. NK cells were isolated from peripheral blood by negative selection using the NK Cell Isolation kit (Miltenyi Biotec, Bergisch Gladbach, Germany) according to the manufacturer's instructions. Experiments were performed when the purity of the NK cells was more than 90%, as determined by FCM.

### Reagents and antibodies

Human recombinant NKG2D-Fc (1299-NK), human recombinant NKp44-Fc, recombinant human ULBP3-Fc, and anti-CD16 (clone 245536) monoclonal antibody were purchased from R&D Systems. FITC-mouse isotype IgG1, PE-mouse isotype IgG1, CD3-PE/CD(16 + 56) -FITC, and CD45-APC were purchased from Beckman. NKG2D-PE (clone 5C6) was purchased from eBioscience. The anti-NKG2D antibody (clone BAT221) was from Miltenyi Biotec. Anti-ULBP3 monoclonal antibodies (clones B2-F1-F1 and B4-C5-D11) were prepared in our laboratory^35^. CD56 microbeads were purchased from Miltenyi Biotech. Pancreatin and FITC-conjugated goat anti-mouse IgG (H + L) (A0568) were purchased from Beyotime, China. Collagenase (C-0130) was from Sigma-Aldrich.

### Transfection with ULBP3-siRNA

ULBP3 expression was knocked down by transfecting cells with ULBP3 siRNA (sc-43006; Santa Cruz Biotech). CD133^−^SW620 cells (5 × 10^5^) were plated in 6-well plates for 18 h and then transfected with 8 μl of ULBP3 siRNA using Lipofector 2000 (Beyotime) in serum free medium for 5 h. Thereafter, the medium was replaced with serum-supplemented medium for 24 h. The cells were then used for functional assays.

### FCM analysis

Cells (1 × 10^5^) were suspended in PBS containing 2% FBS for 10 min to block nonspecific binding sites. To determine the percentage of lymphocyte subsets, the cells were then incubated at 4°C for 30 min with a combination of the following antibodies: NKG2D-PE, ULBP3-FITC, CD3-FITC/CD(16 + 56)-PE, and CD45-APC. For indirect staining, cells were washed twice with PBS and then incubated for 20 min at 4°C with FITC-conjugated goat anti-mouse IgG. Compensation was set with single-stained samples, low forward scatter elements (RBC and debris) were excluded from the analysis, and 10,000 events were collected and analyzed using a FACSAria cytometer (BD Biosciences).

### Polymerase chain reaction (PCR)

The following PCR primers were used: ULBP3, forward 5′- ATTCTTCCGTACCTGCTATT-3′ and reverse 5′-GCTATCCTTCTCCCACTTCT-3′; β-actin, forward 5′-CACGAAACTACCTTCAACTAA-3′ and reverse 5′-CATACTCCTGCTTGCTTGCTGATC-3′. ULBP3 gene expression was normalized to β-actin expression. Total RNA from colorectal tumor cell lines, tumor tissues, and ANTT was extracted using TRIzol after 48 h of cultivation. cDNA was obtained by reverse transcription of mRNA and used as the template for PCR. The PCR program consisted of 95°C for 4 min, followed by 25 cycles of 94°C for 30 s, 57°C for 30 s, and 27°C for 40 s, with a final extension at 72°C for 10 min. All samples were run in triplicate.

### Immunohistochemistry

Colorectal cancer tissues, liver cancer tissues, lung cancer tissues, and gastric cancer tissues were obtained from the department of pathology in The Hospital Affiliated to Jiangsu University. Tissue sections were treated with 0.3% hydrogen peroxidase for 5 min and blocked for 30 min with normal goat serum at room temperature. Anti-ULBP3 monoclonal antibody (B2-FI-FI) (1:200) was applied to the blocked sections and incubated overnight at 4°C. The sections were incubated for 30 min at 37°C with HRP-labeled goat anti-mouse IgG antibody (1:2000), and the signals were developed with diaminobenzidine tetrahydrochloride solution. The sections were viewed with an Olympus Ax-70 DMC Ie CCD camera connected to a PC monitor.

### Cytotoxicity assay

NK cell cytotoxic activity against the tumor cells was determined by measuring the amount of lactate dehydrogenase (LDH) released from the target cells. A commercial LDH cytotoxicity kit (Beyotime) was used according to the manufacturer's instructions. The maximum LDH release was determined by lysing target cells for 30 min using the lysis buffer provided with the assay and measuring LDH in the culture medium. Absorbance for the colorimetric reaction was measured at a wavelength of 490 nm, with a reference wavelength of 655 nm, using a Model 550 microplate reader (Bio-Rad, Hercules, CA, USA). The specific lysis for each effector to target cell (E:T) ratio was calculated with the following formula: % specific lysis = [(experimental release − spontaneous release)/(maximum release − spontaneous release)] × 100.

### Time-resolved fluoroimmunoassay for measuring sULBP3 levels in the serum

The wells of a 96-well plate were coated with the anti-ULBP3 monoclonal antibody B2-F1-F1 (1 μg/well) in coating buffer overnight at 4°C. Human recombination ULBP3-Fc was diluted to different concentrations for use as a standard. Serum samples and tumor cell culture suspensions were added at 100 μl/well and incubated at room temperature with shaking for 1 h and washed 6 times with buffer. Next, Eu^3+^-labeled ULBP3 monoclonal antibody B4-C5-D11 (0.1 ng/well) was added, incubated at room temperature with shaking for 1 h, and washed 6 times with buffer. Accentuation buffer was then added at 100 μl/well, incubated with shaking for 0.5 h, and washed 6 times. The Eu^3+^ fluorescence intensity was measured using an AutoDELFIA 1235 immunoassay system (EG&G Wallac), and data were analyzed using the MultiCalc software.

### Statistics analysis

The results are expressed as the mean ± SEM. Comparisons between 2 groups were performed with Student's *t*-test. Differences among groups were assessed using one-way analysis of variance followed by the Tukey post hoc multiple comparisons test. *P* < 0.05 was considered statistically significant.

## Author Contributions

X.M., Y.Z. and P.J. performed the experiments and analyzed the data. C.M. designed the project and wrote the manuscript. Y.Z. and T.Z. edited various parts of the manuscript. Q.J., X.C., H.L. and T.Z. helped with the experimental design. G.Y. and D.C. supervised the data analysis and edited the manuscript. All authors reviewed the manuscript.

## Figures and Tables

**Table 1:** sULBP3 distribution in serum of cancer patients

	Number	<15 ng/ml (%)	≥15 ng/ml (%)
Colorectal cancer	45	33(73.3)	12(26.7)
Gastric cancer	38	31(83.3)	7(16.7)
Lung cancer	33	28(82.3)	5(17.7)
